# Parallel window decoding enables scalable fault tolerant quantum computation

**DOI:** 10.1038/s41467-023-42482-1

**Published:** 2023-11-03

**Authors:** Luka Skoric, Dan E. Browne, Kenton M. Barnes, Neil I. Gillespie, Earl T. Campbell

**Affiliations:** 1 Riverlane, Cambridge, United Kingdom; 2 Dept. of Physics and Astronomy, University College London, London, WC1E 6BT UK; 3 Dept. of Physics and Astronomy, University of Sheffield, Sheffield, S3 7RH UK

**Keywords:** Qubits, Computer science

## Abstract

Large-scale quantum computers have the potential to hold computational capabilities beyond conventional computers. However, the physical qubits are prone to noise which must be corrected in order to perform fault-tolerant quantum computations. Quantum Error Correction (QEC) provides the path for realizing such computations. QEC generates a continuous stream of data that decoders must process at the rate it is received, which can be as fast as 1 *μ*s per QEC round in superconducting quantum computers. If the decoder infrastructure cannot keep up, a data backlog problem is encountered and the computation runs exponentially slower. Today’s leading approaches to quantum error correction are not scalable as existing decoders typically run slower as the problem size is increased, inevitably hitting the backlog problem. Here, we show how to parallelize decoding to achieve almost arbitrary speed, removing this roadblock to scalability. Our parallelization requires some classical feed forward decisions to be delayed, slowing-down the logical clock speed. However, the slow-down is now only polynomial in the size of the QEC code, averting the exponential slowdown. We numerically demonstrate our parallel decoder for the surface code, showing no noticeable reduction in logical fidelity compared to previous decoders and demonstrating the predicted speedup.

## Abstract (web summary)

In order to be useful for future large-scale quantum computing, quantum error correction needs to allow for fast enough classical decoding time, while at the moment the slowdown is exponential in the size of the code. Here, the authors remove this roadblock, showing how to parallelize decoding and make the slowdown polynomial.

## Introduction

**Fig. 1:**
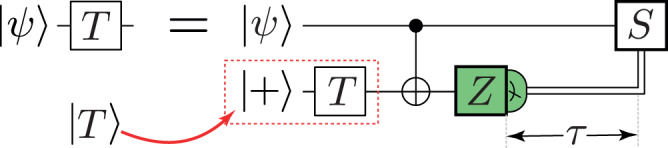
A gate-teleportation circuit to perform a *T* gate using a magic state $\left|T\right\rangle :=T\left|+\right\rangle$, including a classically controlled *S* gate depending on the measurement outcome. In fault-tolerant implementations with logical qubits, the logical *Z* measurement must be decoded before the *S* correction can be correctly applied. This leads to a response time *τ* that is largely determined by the decoding time but also includes communication and control latency.

Quantum error correction (QEC) generates a stream of syndrome data to be decoded. An offline decoder collects and stores all the syndrome data generated during a hardware run (often called a shot) and then performs decoding as a post-processing step. Offline decoding is sufficient for computations consisting solely of Clifford gates (e.g. CNOT and Hadamard gates). However, fault-tolerant quantum computations must adapt in response to certain logical measurement results, which must be decoded to be reliable. For instance, when performing *T* ≔ diag(1, *e*^*iπ*/4^) gates using teleportation and a magic state^1,2^, we must decide whether to apply a Clifford *S* ≔ diag(1, *e*^*iπ*/2^) correction before performing the next non-Clifford operation (see Fig. 1). This logic branching decision can only be reliably made after we decode the syndrome data from the *T* gate teleportation^3–5^. Therefore, online, or real-time, decoding is necessary for useful quantum computation. Classical computation occurs at finite speed, so online decoders will have some latency, but they need only react fast enough to enable feed-forward and Clifford correction.

How fast do decoders need to be? A fundamental requirement was first noted by Terhal^4^ in her backlog argument

“Let *r*_proc_ be the rate (in bauds) at which syndrome bits are processed and *r*_gen_ be the rate at which these syndrome bits are generated. We can argue that if *r*_gen_/*r*_proc_ = *f* > 1, a small initial backlog in processing syndrome data will lead to an exponential slow down during the computation, …”

Terhal proved that quantum algorithms with *T*-depth *k* have a running time lower bounded by *cf*^*k*^ when *f* > 1 and *c* is some constant. Refs. ^6,7^ provide more detailed reviews of this backlog argument.  As we scale the device, for all known decoders the decoding becomes more complex, the value of *f* increases and inevitably we encounter the backlog problem.

Here we solve this problem, removing a fundamental roadblock to scalable fault-tolerant quantum computation. We propose parallelized window decoding that can be combined with any inner decoder that returns an (approximately) minimum weight solution, presenting results for minimum-weight perfect matching (MPWM)^8–10^ and union-find (UF)^11,12^.

The previous leading idea to modify decoders to work online was proposed by Dennis et al.^8^:

“…take action to remove only these long-lived defects, leaving those of more recent vintage to be dealt with in the next recovery step.”

Here defects refer to observed changes in syndrome. Dennis et al. called this the overlapping recovery method^8,13^. Later, similar approaches were adopted for decoding classical LDPC codes^14^, where this is known as sliding window decoding. Roughly speaking, given a sequence of defects proceeding in time one decodes over some contiguous subset, or window. The decoder output gives only tentative error assignments, and from these only a subset—those of an older vintage—are committed. Here, committing means making a final correction decision for potential error locations, with all corrections performed in software. One then slides the window up and the process repeats.

Sliding window decoding is inherently sequential. Let us consider a single code block (e.g. a surface code patch) with each QEC round taking *τ*_rd_ seconds. If each window is responsible for committing error corrections over *n*_com_ rounds of syndrome data, then it takes time *n*_com_*τ*_rd_ to generate all this data. If the time to decode each window is *τ*_W_, including any communication latency, then avoiding Terhal’s backlog problem requires that *τ*_W_ < *n*_com_*τ*_rd_. Since *τ*_W_ typically grows superlinearly with the decoding volume, this leads to a hard upper bound on the achievable distance *d*. For example, a distance *d* surface code has *τ*_W_ = Ω(*n*_com_*d*^2^) and therefore we are restricted to *d*^2^ ≤ *O*(*τ*_rd_). Scaling hardware based on a fixed device physics means *τ*_rd_ is fixed. This imposes a hard limit on code distance. The reader should pause to reflect how remarkable it is that the current leading proposal for fault-tolerant quantum computation is not scalable.

As with sliding window decoding, our parallel window decoder breaks the problem up into sets of overlapping windows. Rather than solving these sequentially, some windows are decoded in parallel by adapting how overlapping windows are reconciled. Through numeric simulations, we find that sliding, parallelized and global approaches differ in logical error rates by less than the error bars in our simulations. We show that, by scaling classical resources, the parallel window can achieve almost arbitrarily high *r*_proc_ regardless of decoding time per window *τ*_W_. Furthermore, we show that while there is still an inherent latency determined by *τ*_W_ leading to a slow-down of the logical clock speed, this is only linear in *τ*_W_, rather than the exponential slowdown resulting from Terhal’s backlog argument. We conclude with a discussion of the implications of this work for practical decoder requirements and extensions to a number of other decoding problems. After making this work public, similar results were posted by the Alibaba team^15^. The Alibaba numerics present the logical fidelity of the decoder, but do not include numerical results on decoding speed and improvements through increasing number of processors used.

Single-shot error correction is a different paradigm of decoding that uses only the results of a single round of QEC measurements, without any historical data. This type of decoding is only possible for certain quantum codes, such as higher-dimensional topological codes^16–19^ and quantum low-density parity-check codes (qLDPC)^20,21^. To date, no such code has yet been able to reproduce the very high threshold of the surface code. Furthermore, single-shot error correction is still susceptible to a backlog problem. In every analysis of single-shot QEC, it has been assumed that the correction for previous QEC rounds has already been performed before the next round of decoding is performed. However, if these decoding problems take longer to solve than the time to perform a round of QEC, then even single-shot QEC encounters a backlog problem. This situation is worse when the backlog problem is encountered by a single-shot decoder, because it cannot be alleviated by using the single-shot decoder in conjunction with the parallel decoding methods proposed here.

## Results

### Matching decoders

Windowing techniques, both sliding and parallel, can be combined with most decoders acting internally on individual windows. We will refer to these as the inner decoders. The only property we assume of the inner decoder is that it returns a correction that is (approximately) the lowest weight correction. We otherwise treat the inner decoder as a black box. For brevity, in the main text we will describe the procedure for the case of matching decoders, such as MWPM and UF. A matching decoder is applicable when any error triggers either a pair of defects or a single defect. For example, in the surface code *X* errors lead to pairs of defects (when occurring in the bulk) or a single defect (when occurring at so-called rough boundaries of the code). To fully formulate a matching problem, all errors must lead to a pair of defects. Therefore, errors triggering a single defect are connected to a virtual defect commonly called the boundary defect. We then have a graph where the vertices are potential defects (real or boundary) and edges represent potential errors. Given an actual error configuration, we get a set of triggered defects and we can enforce that this is an even number by appropriately triggering the boundary defect. A matching decoder takes as input this set of triggered defects and then outputs a subset of edges (representing a correction) that pair up the triggered defects. Running a decoder on our entire defect data set at once (no windowing) will be referred to as global decoding, but global decoding is not compatible with the real-time feedback required for non-Clifford gates.

### Sliding window decoding

Instead of decoding a full history of syndrome data after the computation is complete, sliding window decoding starts decoding the data in sequential steps while the algorithm is running. At each step, a subset (a window) of *n*_W_ rounds of syndrome extraction is processed. The window correction graph is acquired by taking all the vertices and edges containing defects in the selected rounds. The measurement errors in the final round of a window only trigger a single defect within the window. Therefore, all final round defects are additionally connected to the boundary defect, referred to as the rough top time boundary.

Following the overlapping recovery method^8,13^, a window can be divided into two regions: a commit region consisting of the long-lived defects in the first *n*_com_ rounds, and a buffer region containing the last *n*_buf_ rounds (*n*_W_ = *n*_com_ + *n*_buf_). An inner decoder (e.g. MWPM or UF) outputs a subset of tentative correction edges within the window. Only the correction edges in the commit region are taken as final. Sometimes, the chains of tentative correction edges will cross from the commit to the buffer region. Applying only the part of the chain in the commit region will introduce new defects, referred to as artificial defects along the boundary between the commit and buffer regions.

**Fig. 2:**
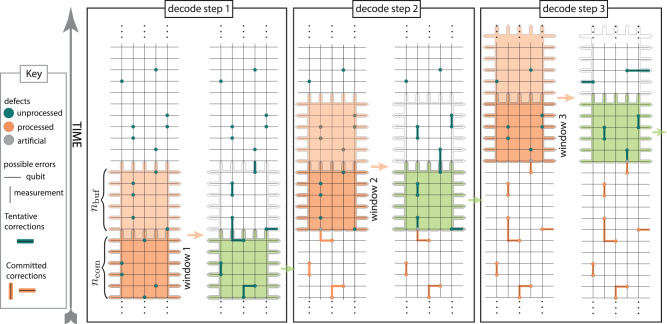
Sliding window decoding schematic for 2D decoding problem, for example representing the repetition code with phenomenological noise. At each decoding step a number of syndrome rounds (window) is selected for decoding (orange region in left columns), and tentative corrections acquired. The corrections in the older *n*_com_ rounds (green region in right columns) are of high confidence and are committed to, while the corrections in the remaining (buffer) *n*_buf_ rounds are discarded. The window is then moved up to the edge of the commit region and the process repeated. We decide to commit to the edges going from the commit region out of it, producing artificial defects defined by nodes outside of the region belonging to such an edge. All numerics performed using a generalisation of this method to the 3D decoding problem representing the surface code with circuit-level noise.

The window is then moved up by *n*_com_ for the next decoding step that now includes the artificial defects along with the unresolved defects from the buffer region of the preceding step and new defects in the following rounds. Figure 2 illustrates sliding window for the simple example of a repetition code, naturally extending to surface codes by adding another spatial dimension. Notice in Fig. 2 the creation of artificial defects where tentative corrections cross between commit and buffer regions.

Due to these artificial defects, sliding window decoding (and also parallel window decoding, described below) requires an inner decoder, which returns an approximately low weight correction, such as UF or MWPM. Decoders, such as those based on tensor network contractions, identify the optimal homology class (all errors strings corresponding to contractible loops are in the same class) that contains a low-weight correction. Once a homology class has been identified, we can always efficiently select a representative correction from the class but this could be a high-weight correction (e.g. containing many contractible loops), leading to additional artificial defects at the boundary of the committed region, and then to logical errors when the next window is decoded. Therefore, additional modifications beyond those discussed in this work would be needed to use homology-based inner decoders.

Processing only a subset of the syndrome data at a time inevitably reduces the logical fidelity of the decoder. However, a logical fidelity close to that of the global decoder can be retained by making the unaccounted failure mechanisms negligible compared to the global failure rate. In particular, the error chains beginning in the committed region need to be unlikely (compared to the global failure rate) to span the buffer region and extend beyond the window. If the measurement and qubit error rates are comparable, to achieve this for distance *d* codes, it suffices to make the buffer region of the same size *n*_buf_ = *d*^8^. In the Supplementary Note Section 3, we demonstrate numerically that by choosing *n*_buf_ = *n*_com_ = *d* there is no noticeable increase in logical error rate when applying the sliding window algorithm. Indeed, in our numerics we saw some evidence that one can use *n*_buf_ < *d* without significant degradation, provided *n*_buf_/*d* remains sufficiently large, though we do not thoroughly investigate this in detail here.

### Parallel window decoding

**Fig. 3:**
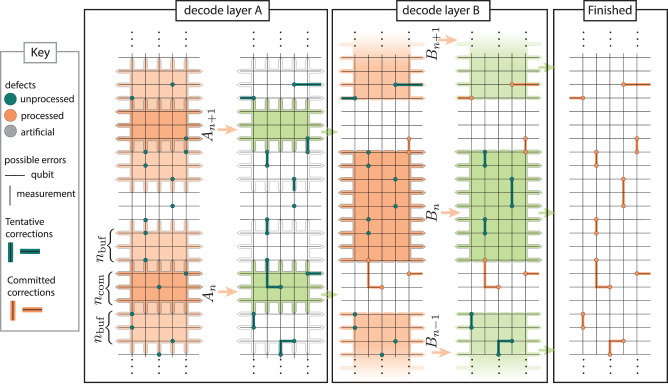
Parallel window decoding schematic for 2D decoding problem, for example representing the repetition code with phenomenological noise. The decoding proceeds in two layers. In layer A, a number of non-overlapping windows *A*_*n*_ is decoded in parallel. The high confidence corrections in the middle *n*_com_ rounds of each window are committed to, while the corrections in the surrounding *n*_buf_. The artificial defects are passed on to layer B. Windows *B*_*n*_ in layer B are fully committed to, resolving all the defects between the committed regions of layer A and completing the correction. All numerics are performed using a generalisation of this method to the 3D decoding problem representing the surface code with circuit-level noise.

Here we present our main innovation to overcome the backlog problem, which we call parallel window decoding. We illustrate the method in Fig. 3. As in Fig. 2, our illustration is for a repetition code example. These figures are for illustrative purposes only, with all numerical results using the natural generalization to the 3D decoding problem of the surface code (further extensions discussed in Section III).

Parallel window decoding proceeds in two layers. First, we process a number of non-overlapping windows in decode layer A concurrently. As opposed to the sliding window approach, there are potentially unprocessed defects preceding the rounds in an A window. We thus need to include a buffer region both preceding and following the commit regions. Additionally, we set both time boundaries to be rough, connecting the first and last round of defects to the boundary node. We set *n*_buf_ = *n*_com_ = *w*, giving a total of *n*_W_ = 3*w* per window for some constant *w*. Using the same reasoning as with the sliding window we set *w* = *d*. Note that in Fig. 3 we use *w* < *d* to keep the illustration compact.

Having committed to corrections in adjacent windows and computed the resulting artificial defects, in layer B we fill in the corrections in the rounds between the neighbouring A commit regions. For convenience, we separate A windows by *d* rounds, so that B windows also have *n*_W_ = 3*d* rounds. As we have already resolved the nearby defects preceding and succeeding each B windows, the B windows have smooth time boundaries and do not require buffers.

**Fig. 4:**
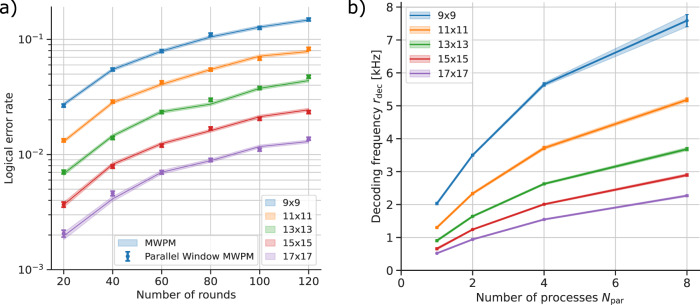
Logical error rate and decoding frequency on a *d* × *d* × *N*_rounds_ rotated planar code using Minimum Weight Perfect Matching (MWPM) under circuit-level noise with *p* = 0.5% (see Methods). **a** Logical error rates as a function of the number of rounds of syndrome extraction for different code sizes for both the global offline MWPM (shaded bands), and using the parallel window algorithm (points). The parallel window decoder has no numerically significant drop in logical fidelity compared to the global decoder. Additional data with a different noise rate *p* and using phenomenological noise is presented in Supplementary Figures 2 and 3. **b** The decoding frequency (number of rounds decoded per second) as a function of the number of decoding processes for the parallel window algorithm. The decoding frequency increases with the number of processes, achieving approximately linear speed-up with the number of processes for harder decoding scenarios (*d* ≥ 15). The sub-linearity most noticeable on small decoding problems is due to the parallelization overhead in the software implementation. Error bars represent standard deviation over samples. Where the error bars are not visible, they are smaller than the marker size. Here we plot the decoding frequency *r*_dec_, therefore the rate of syndrome processing is *r*_proc_ = *r*_dec_(*d*^2^ − 1).

Crucially, if the size of buffer region in layer A is chosen appropriately, we expect no significant drop in logical fidelity compared to the global decoder. As with sliding windows, this is because each error chain of length ≤*d* is guaranteed to be fully captured within one of the windows. In Fig. 4a we verify this by simulating the decoding process on a *d* × *d* × *N*_rounds_ rotated planar code under circuit-level noise (see Methods for the noise model details). We find that the logical error rates of rotated planar codes using the global MWPM and parallel window MWPM are within the numerical error of each other across a range of code sizes and number of measurement rounds. The same holds for UF-based decoders, as well as different noise models, with the data presented in the Supplementary Note Section 3.

This approach is highly parallelizable: as soon as the last round of window *A*_*n*_ has been measured, the data can be given to a worker process to decode it. However, as the window *B*_*n*_ requires the artificial defects generated by windows *A*_*n*_ and *A*_*n*+1_ adjacent to it (see Fig. 3), it can only start once both processes have completed. In the Supplementary Figure 4, we sketch a schematic defining how the data pipelining could be implemented in an online parallel window decoder to achieve a high utilization of available decoding cores.

Assuming no parallelization overhead, the syndrome throughput will scale linearly with the number of parallel processes *N*_par_. In this case, *N*_par_*n*_com_ rounds are committed to in layer A, and *N*_par_*n*_W_ in layer B. Each round takes *τ*_rd_ to acquire and the two layers of decoding take 2*τ*_W_. To avoid the backlog problem, we need the acquisition time to be greater than the decoding time:

1 $${N}_{{{{{{{{\rm{par}}}}}}}}}({n}_{{{{{{{{\rm{com}}}}}}}}}+{n}_{{{{{{{{\rm{W}}}}}}}}}){\tau }_{{{{{{{{\rm{rd}}}}}}}}}\ge 2{\tau }_{{{{{{{{\rm{W}}}}}}}}}.$$

Therefore, the number of processes needs to be at least:

2 $${N}_{{{{{{{{\rm{par}}}}}}}}}\ge \frac{2{\tau }_{{{{{{{{\rm{W}}}}}}}}}}{({n}_{{{{{{{{\rm{com}}}}}}}}}+{n}_{{{{{{{{\rm{W}}}}}}}}}){\tau }_{{{{{{{{\rm{rd}}}}}}}}}}.$$

In practice, the overhead of data communication among worker processes needs to be considered. In the parallel window algorithm, each process only needs to receive defect data before it is started, and return the artificial defects and the overall effect of the committed correction on the logical operators (see the Supplementary Note Section 4). Thus, we expect the data communication overhead to be negligible compared to the window decoding time. Indeed, in Fig. 4b we demonstrate this by simulating parallel window decoding in Python using MWPM as the inner decoder, showing how using *N*_par_ = 8 leads to nearly an 8x increase in decoding speed. Some sub-linearity can be seen due to parallelization overheads in software, particularly for low-distance codes where the decoding problem is relatively simple. In the Supplementary Note Section 3, we repeat these simulations using UF decoder where the overhead is more noticeable due to faster decoding of individual windows. However, hardware decoders such as FPGA (Field Programmable Gate Array) and ASIC (Application-Specific Integrated Circuit) are more suited to parallel data processing, allowing a large number of processes without being bottle-necked by the communication overheads (discussed further in the Supplementary Note Section 4). Lastly, even with some sub-linearity, the backlog can be averted provided arbitrary decoding speed is achieved with a polynomial number of processors.

## Discussion

While we can achieve almost arbitrarily high syndrome processing rates, there is still an inherent latency determined by the time to decode each window *τ*_W_. If *τ*_W_ is large compared to the physical QEC round time *τ*_rd_, we may slow down the logical clock of the quantum computer to compensate for this latency. This slowdown is achieved simply by extending the delay time *τ* as shown in Fig. 1. If we pick *N*_par_ as described in Eq. (2), at every instance a block of *n*_lag_ = *N*_par_(*n*_com_ + *n*_W_) rounds are being decoded at once. The last round for which the full syndrome history has been decoded is therefore going to be *n*_lag_ rounds behind the most recently measured syndrome data. Therefore, we can set the response time after each *T*-gate (as defined in Fig. 1) to

3 $$\tau={n}_{{{{{{{{\rm{lag}}}}}}}}}{\tau }_{{{{{{{{\rm{rd}}}}}}}}}={N}_{{{{{{{{\rm{par}}}}}}}}}({n}_{{{{{{{{\rm{com}}}}}}}}}+{n}_{{{{{{{{\rm{W}}}}}}}}}){\tau }_{{{{{{{{\rm{rd}}}}}}}}}$$

However, combining Eq. (2) and Eq. (3) the total response time is ≈ 2*τ*_*W*_. That is, for an algorithm with *k* layers of *T* gates, the total response time is *τk* ≈ 2*kτ*_*W*_. This is in stark contrast to the exponential in *k* response time observed by Terhal^4^. Furthermore, using an efficient decoder for each window, the average window decode time *τ*_*W*_ scales polynomially with code size *d*, so *τ*_*W*_ = *O*(*d*^*α*^) for some constant *α*. Since code size is poly-logarithmic in algorithm depth *k* and width *W*, $d=O(\log {(kW)}^{\beta })$ for some constant *β*. The response time per layer of *T*-gates is a poly-logarithmic factor so $\tau=O(\log {(kW)}^{\alpha \beta })$. Strictly speaking, this additional overhead increases the decoding volume *kW* by a logarithmic factor, but overall still gives a poly-logarithmic complexity.

We define logical clock time as how long it takes to execute one logical non-Clifford gate. Using lattice surgery to perform *T*-teleportation — and assuming no bias between measurement and physical errors — it takes *dτ*_rd_ time for lattice surgery and *τ* response time. This gives a logical clock time of *τ*_clock_ ≔ *dτ*_rd_ + *τ*. Alternatively, this time overhead can be converted into a qubit overhead by moving Clifford corrections into an auxiliary portion of the quantum computer^22^, for example using auto-corrected *T*-gate teleportation^2,23^. In algorithm resource analysis, a common assumption is that *T* gates are performed sequentially ^2,24–31^ as then only a few magic-state factories are needed to keep pace. Auto-correction gadgets enable us to perform the next *T*-gate before the response time has elapsed. The price is that an auxiliary logical qubit must instead be preserved for time *τ*, after which it is measured in a Pauli basis depending on the outcome of the decoding problem. Therefore, instead of a time overhead we can add ⌈*τ*/*dτ*_rd_⌉ auxiliary logical qubits. If we have an algorithm with 100 logical qubits and *τ*_clock_ = 10*dτ*_rd_, then: without autocorrection we incur a 10 × time cost; and with autocorrection we instead require 9 auxiliary logicals qubits and so a 1.09 × qubit cost. Under these common algorithm resource assumptions, we the find seemingly large time overheads from parallel window decoding can be exchanged for modest qubit overheads. Indeed, the auto-correction strategies trade time for space resource, but the overall space-time volume is preferable under these resource estimation assumptions (1.09 × instead of 10 × ). Note that the additional space-time volume required for magic state distillation will depend only on the number of magic states produced and not on whether we use auto-corrected teleportation.

Our proposed decoder admits several extensions. Error mechanisms (e.g. *Y* errors in the bulk of the surface code) sometimes trigger more than a pair of defects, but reasonable heuristics can often be used to approximately decorrelate these errors to produce a graphical decoding problem. This decorrelation works well for the surface code. However, many codes cannot be decorrelated and require a non-matching decoder. Even when decorrelation approximations are possible, logical fidelities can be improved by using a non-matching decoder that accounts for this correlation information^32–35^. Extensions of parallel window decoding to non-matching inner decoders are outlined in the Supplementary Note Section 2.

By judicious choice of window shapes and boundaries, one could consider 3D-shaped windows that divide the decoding problem in both space and time directions. Similarly, we can construct 3D-shaped windows for parallel execution with only a constant number of layers. When slicing in the time direction we only needed 2 layers of windows, but when constraining window size in *D* dimensions a *D* + 1 layer construction is possible, with the minimum number of layers being determined by the colorability of some tiling (see the Supplementary Note Section 1 for details). When performing computation by lattice surgery, during merge operations the code temporally has an extended size^2,27,36,37^, and windowing in the spatial direction will become necessary to prevent the window decode time *τ*_*W*_ from significantly increasing. One may also wish to spatially window for a single logical qubit with windows smaller than the code distance since the decoder running time *τ*_*W*_ reduces with window size, and therefore the logical clock time may decrease (alternatively auto-correction qubit overhead may reduce). But there are subtle tradeoffs. For windows of size *ω* < *d* in either the space or time direction, there may be adversarial failure mechanisms of weight (*ω* + 1)/2 < (*d* + 1)/2 that are no longer correctly decoded. One may speculate that this reduces the effective code distance to *ω*. However, in practice, percolation theory arguments^38^ show that for a distance *d* code, the largest error clusters are typically of size $O({{{{{{{\rm{polylog}}}}}}}}(d))$. This leaves open the possibility that windows of size $O({{{{{{{\rm{polylog}}}}}}}}(d)) \, < \, \omega \, < \, d$ will suffice and be of practical value for stochastic (even if not adversarial) noise, though substantial further investigation is required. We remark that this discussion assumes that measurement errors (that create vertical error chains) have a comparable probability as physical Pauli errors. If there is a large measurement error bias, then we must appropriately scale the duration of lattice surgery operations and the vertical extent of our windows.

In summary, parallel window decoding avoids the exponential backlog growth that is unavoidable (for large enough computations) with sliding window decoders. For many leading hardware platforms, such as superconducting devices, syndrome backlog can be a severe practical obstacle, even for modest code sizes. In recent superconducting experiments a QEC round was performed every 1.1 *μ*s by Krinner et al.^39^ and every 921 ns by the Google Quantum AI team^40^. Our results are applicable to all hardware platforms, but the speed of superconducting quantum computers means these are amongst the most challenging systems for real-time decoding. Indeed, both aforementioned teams instead performed offline decoding, omitting a crucial aspect of scalable error correction.

To meet this challenge, improving the speed of decoders is currently an area of intense research. For example, LILLIPUT^41^ is a recently proposed fast online sliding window decoder, implemented as an FPGA-based look-up table. For *d*≤5 surface codes, the authors reported that a round of syndrome data could be processed every 300 ns, fast enough even for superconducting qubits. However, the memory requirements of lookup tables scale exponentially in qubit number, making this decoder impractical for all but the smallest code sizes. The UF decoder scales favourably, and modelling of it on a dedicated microarchitecture^12^ suggested it would be fast enough for distance 11 surface codes. However, the authors acknowledged that: “further study is necessary to confirm the validity of our model in a real device". Riverlane recently showed real-time FPGA decoding of single windows fast enough to beat the backlog problem without any further parallelization up-to-distance 21 codes^42^. There have been other approaches to accelerating decoders. A parallelized version of minimum weight perfect matching (MWPM) has been proposed^43^ but never implemented and its performance is unclear. Adding a predecoding stage has also been identified as a way to further accelerate decoding and potentially boost logical fidelity^7,44–48^, but this has not been tested in an online setting. As such, for larger code distances, it is unclear whether conventional decoding approaches will be fast enough.

On the other hand, a parallel window decoder, as introduced here can achieve almost arbitrarily high decoding speed given enough classical resources and some (polynomially scaling) quantum resource overheads. Therefore, this approach resolves both fundamental scalability issues and practical obstacles for hardware with rapid QEC cycle times.

## Methods

All simulations were performed on a  standard D32as v4 Azure instance. We used the PyMatching package^10^ to perform MWPM. For UF we used a custom Python implementation of the algorithm described in Ref. ^11^.

The decoding graph for each window is acquired by taking a subset of edges between nodes belonging to vertices in the window. Depending on the rough bottom (top) time boundaries, we further add edges connecting each of the nodes in the first (last) round to the boundary node. These are assigned a probability equal to the probability of triggering one of the edges connecting the given node in the window to a node out of the window.

In all experiments, we compute the logical error rate of sliding and parallel window methods for rotated planar code with different code sizes and increasing number of decoding rounds. We use circuit-level noise parametrized by *p* = 0.5% with the following noise model:

- *p* two-qubit depolarising noise after each two-qubit gate
- measurement results flipped with probability *p*
- *p*/10 depolarising noise after each single-qubit gate and reset operation
- *p*/10 depolarising noise for any idling qubit while gates are applied elsewhere

The syndrome data for the noise model has been sampled using Stim simulation package^49^.

To compute the timing for Fig. 4b, we perform the decoding on $(4\max {N}_{{{{{{{{\rm{par}}}}}}}}}+1)d$ rounds to ensure a full utilization of parallel resources if both A and B decoding steps. We assume initialisation and readout in the Z basis, meaning that the initial and final rounds of defects are smooth. Moreover, in parallel window decoding, we take the first round to always belong to layer A, and the first 2*d* rounds of the first window are committed to. The last round belongs to a layer B if the total number of rounds *n*_tot_ satisfies ${n}_{{{{{{{{\rm{tot}}}}}}}}}\,{{{{{{\mathrm{mod}}}}}}}\,\,4d\in (-d,d]$, in which case the decoding is performed normally with the last B window potentially being of reduced size. Otherwise, the last window belongs to layer A and the commit region of the last window is from the bottom of the regular commit region to the last round.

### Supplementary information

Supplementary Information

Peer Review File

## Data Availability

The stim circuits and data that support the findings of this study are available in Zenodo with the DOI identifier 10.5281/zenodo.8422904.
