# Supplementary material for: Parallel window decoding enables scalable fault tolerant quantum computation
Source: Nat Commun. 2023 Nov 3;14:7040. doi: 10.1038/s41467-023-42482-1 (PMC10624853; doi:10.1038/s41467-023-42482-1)
Supplement: Supplementary file 1 — Supplementary Information [file 41467_2023_42482_MOESM1_ESM.pdf]

Supplementary information for: Parallel window decoding enables scalable fault tolerant quantum computation

Luka Skoric,[1] Dan E. Browne,[1,2] Kenton M. Barnes,[1] Neil I. Gillespie,[1] and Earl T. Campbell[1,3]

[1]*Riverlane, Cambridge, United Kingdom*

[2]*Dept. of Physics and Astronomy, University College London, London, WC1E 6BT, UK*

[3]*Dept. of Physics and Astronomy, University of Sheffield, Sheffield S3 7RH, UK*

(Dated: October 6, 2023)

This document provides supplementary material to the article: "Parallel window decoding enables scalable fault tolerant quantum computation"

Contents

1. Parallel window decoding in time and space 1

2. Non-matching inner decoders 2

3. Numerical validation of decoder performance 3

4. Decoding pipeline 4

References 7

Supplementary Note 1 – Parallel window decoding in time and space

Our main argument has centered around how to perform parallel window decoding over windows defined by time intervals. However, as motivated in the main text, we also may want to parallelize with respect to spatial directions. This is required to support long range lattice surgery operations [1–5], and may also be desirable within single patches. Here we outline how this works, with a guiding example given in Supplementary Figure 1.

First, given some space (e.g. a decoding graph or hypergraph) we divide the space up into non-overlapping commit regions. We regard each vertex in the decoding problem as having a space-time coordinate in $\mathbb{R}^D$ (with $D = 2$ for the surface code). Each edge in the decoding graph is assigned a space-time coordinate corresponding to the mid-point between the vertices it connects. For edges connecting to the boundary, we can just equate the non-boundary vertex coordinate with the edge coordinate. Then for any space-time region, we can associate a set of vertices and edges residing within this region. Assuming a topological code that has local stabilizers, then will always be a maximum distance R between any pair of vertices connected by an edge.

Therefore, to find a valid ordering of layers, it suffices to solve a colouring problem. That is, we define collections of commit regions and seek to assign them colours, such that (i) no two regions of the same colour are adjacent; (ii) length scales are set so that regions of the same colour are always separated by distance R. Given such a colouring, we can map colours to decoding layers, for example red $\rightarrow A$, green $\rightarrow B$ and blue $\rightarrow C$. Any permutation of layers remains a valid choice.

We can regard commit regions A and B of the parallel window method as representing a 2-colouring of a 2D space (see Fig. 3 in the main text). This is extended to 3D (and thereby the surface code decoding problem) by extruding into a 3rd dimension. Supplementary Figure 1-i shows a hexagonal 3-colouring of a 2D space, and Supplementary Figure 1-iii shows the extruded 3D version of this tiling. For a D dimensional space there exist tilings that can be coloured using $D + 1$ colours with each tile of bounded size, which for instance has been proved in the context of colour codes [6, 7]. In Supplementary Figure 1-iii, we tile a $D = 3$ space using only 3 colours, but the regions are unbounded size with respect to depth in the 3rd dimension. If we desire constant size tiles, then a tiling of 3D space could be achieved using 4 colours.

Our examples show the minimum number of colours. Given a limited number of processors N_{par}, we may choose to use more colours so that for each colour there are no more than N_{par} regions.

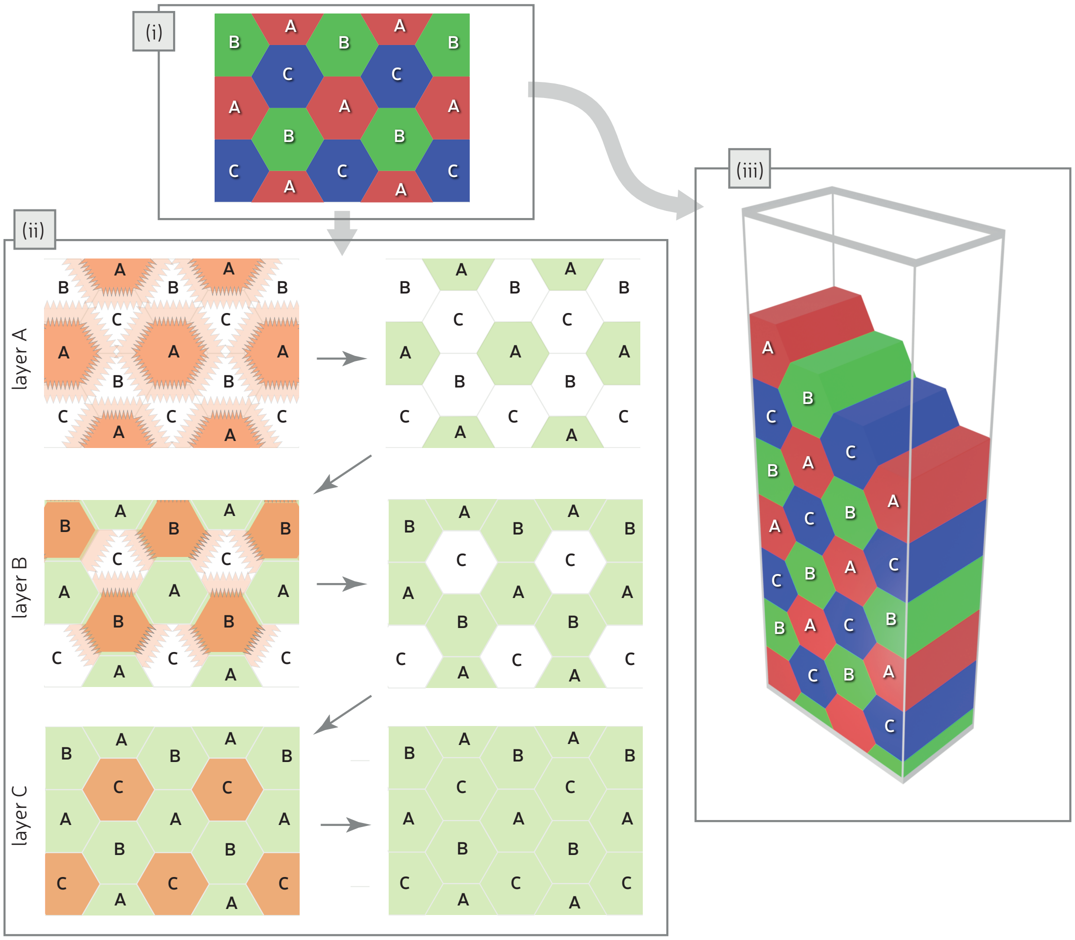

Supplementary Figure 1. Parallel window decoding in both time and 1 spatial dimension and the relationship to colourability of tessellations. (*i*) A 3-colour hexagonal tessellation of a 2D space, with each colour assigned a layer label A, B or C. Note that hexagons of the same colour never touch. (*ii*) A protocol (in 2D) based on the hexagonal tiling. The colours here match those used in Fig. 3 in the main text. That is, dark orange indicates a commit region and light orange shows the buffer region. Zig-zag boundaries represent rough boundaries. Green indicates regions where all the defects have been resolved. (*iii*) The hexagonal pattern of (*i*) extruded into the 3rd dimension, so it is suitable for surface code decoding (e.g 2D+1 decoding problems).

Next, we consider the buffer regions required to provide confidence in the corrections in the commit regions. The buffer windows are placed above and below the commit region of layer A (see Fig. 3 in the main text). In higher dimensions, the buffer regions must include all possible error locations (edges) within a distance w of the commit region. However, previously committed regions must not be included in the construction of buffers. Additionally, we do not want artificial defects pushed into a previously resolved region. Therefore, where a window meets a previously committed region the boundary must be set to smooth (no artificial defects allowed).

For example, Supplementary Figure 1-ii shows buffer regions and boundaries for a hexagonal tiling. In layer A, the buffer region extends in every direction from the commit region. All the boundaries in A are rough. In layer B, the buffer extends in all directions except those already resolved in layer A. Furthermore, the layer B window boundaries are set rough except where they meet the resolved layer A commit regions (where they are instead smooth, as illustrated). The final layer C will only have smooth boundaries and no buffer regions.

Supplementary Note 2 – Non-matching inner decoders

Non-matching decoding problems arise when we also include the possibility of hyperedges. That is, given an error E, the associated hyperedge is a list of all the defects it triggers should the error occur. If this list of defects contains

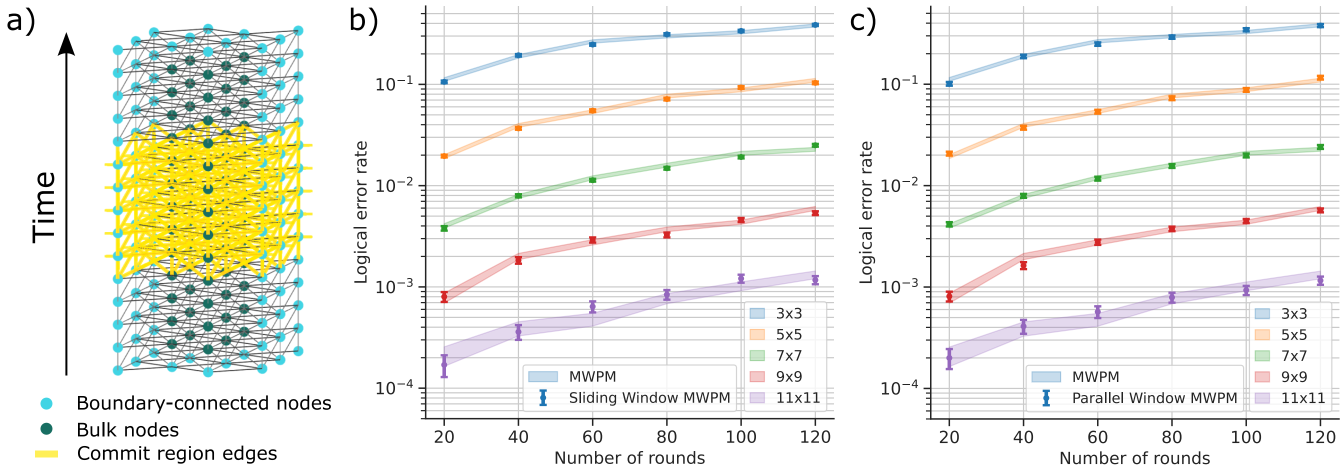

Supplementary Figure 2. Windowed decoding with circuit-level noise. (a) An example of a decode layer A window graph with circuit-level noise on distance 5 rotated planar code. The commit region edges are marked in yellow. (b, c) Logical error rates as a function of the number of rounds of syndrome extraction for different code sizes under 0.2% circuit-level noise for the global MWPM (lines), and using the (b) sliding and (c) parallel window MWPM decoder (points). Error bars represent standard deviation over samples.

more than 2 elements, we say it is a hyperedge. To extend our method, we simply partition all the hyperedges into sets that we call commit regions. Two commit regions can be of the same colour (and therefore part of the same layer) provided that there is no vertex/defect contained in hyperedges from both sets.

This partitioning can be performed either by defining a distance metric derived from the hypergraph, or by defining space-time regions and with each hyperedge having a space-time co-ordinate based on the mid-point of its associated vertices. Note that for non-topological codes the decoding hypergraph may not be localized in Euclidean space, though repeated syndrome extraction means that there will be a time axis such that hyperedges contain vertices that are contained within a constant range on the time axis.

For buffer regions, we follow the same recipe as in the matching case. The difference between rough and smooth boundaries needs additional care. Wherever we have a rough boundary (extremal hyperedges in a buffer region that are not adjacent to any previously corrected/committed regions), we need to allow for the possibility of creating artificial defects. This can be achieved by connecting every hyperedge on a rough boundary to the boundary vertex.

Supplementary Note 3 – Numerical validation of decoder performance

In the main text, we presented numerical results for parallel window decoding using a MWPM inner decoder. Here we present and discuss some additional numerical results: additional circuit-level noise data for both sliding and parallel window logical error rates; the performance of sliding window decoders with a MWPM inner decoder; and parallel window decoding with a UF inner decoder.

In Supplementary Figure 2 we present sliding and parallel window logical error rate data for $p = 0.2\%$ circuit-level noise. Similarly as in the main text, we do not see a noticeable difference in logical error rates between the windowed approaches and the global decoder.

In Supplementary Figure 3 we perform similar simulations using a phenomenological Pauli noise with physical error rate p, meaning that there is a probability p for a data error on every qubit at each round. Further, every syndrome measurement had an error with probability p. In Supplementary Figure 3a we confirm that the sliding window decoding has a negligible drop in logical fidelity for $n_{\mathrm{W}} = 2d$, $n_{\mathrm{com}} = d$ when compared to the global MWPM decoder. Furthermore, in Supplementary Figure 3b we measure the decoding frequency as a function of code size for square rotated planar codes. As the code size grows, the decoding frequency is expected to reduce as $O(1/\mathrm{poly}(d))$, which is consistent with our data. Therefore, using sliding window decoding combined with any of the leading inner decoding algorithms, there will always be a code distance for which $\tau_{\mathrm{W}} > n_{\mathrm{com}}\tau_{\mathrm{rd}}$. This sets a limit on the distance up to which error correction codes can scale using sliding window decoding.

Next, we discuss parallel window decoding under phenomenological noise comparing both UF and MWPM inner decoders (Supplementary Figure 3c,e. As with MWPM, we see no significant increase of the logical error rate when

using parallel window decoding, and a roughly linear increase with the number of processes N_{par} for large codes (Supplementary Figure 3d,f). However, in the case of smaller codes and faster UF decoder (Supplementary Figure 3d), the decoding problem is relatively easy and we see diminishing returns with increased parallelism as the parallelization overheads in Python start being comparable with the decoding time of individual windows.

Sending data to a worker process, starting the decoding of a window and receiving the resulting data takes a finite amount of time τ_0. Therefore, if $N_{\text{par}}\tau_0 > \tau_{\text{W}}$ all parallel processes will never be fully utilized and the processing will be bottle-necked by these overheads. However, in a hardware decoder, we expect τ_0 to be below 10 ns using modern hardware and syndrome compression techniques [8], allowing us to scale to over 100 processes. As separate processes do not need to share data, further parallelization of data communication is possible, allowing for even higher bandwidths.

In order to decode large scale quantum computation with $O(10^{12})$ gates, we need code sizes of approximately distance 30. The recent advances in highly optimized decoders such as PyMatching 2 have demonstrated the ability to decode distance 30 codes in under 10 μs for $p = 0.1\%$ circuit-level noise [9]. Considering 1μs per QEC round superconducting hardware and barring further advances in decoding algorithms, we therefore believe that $O(10)$ parallel windows would be sufficient to decode large-scale fault-tolerant quantum computers.

Supplementary Note 4 – Decoding pipeline

In Supplementary Figure 4, we sketch the data-flow of the parallel window decoder with $2n$ processes that could be implemented in hardware. As the stream of syndrome data is acquired, it is given to the process manager that is in charge of passing the data to the appropriate decoding block. Each decoding block resolves the $3d$ rounds of defects given to it using a matching decoder of choice, and a given specification of rough time boundaries. Recall that the decode windows are labelled A_i and B_i respectively, where i is an integer. In the k-th step, DA_i (DB_i) blocks decode windows A_{kn+i} (B_{kn+i}) which have rough (smooth) time boundaries (see Fig. in the main text). The exception are the first and the last blocks of computation whose boundaries depend on the initialized state and the basis of measurement.

When the first $3d$ rounds have been collected, these are sent to block DA_0 for decoding, together with the bottom boundary-type information. The next d rounds are given to DB_0 block which has to wait for DA_0 and DA_1 to finish before starting, followed by $3d$ rounds for block DA_1 and continuing until all blocks are running. Once DA_i finishes decoding, it sends the artificial defects and unresolved syndromes from the bottom d rounds to DB_{i-1}, and from the top d rounds to DB_i. The indices are cyclic with period n, meaning that $DB_{-1} = DB_{n-1}$, and DA_{n-1} block is followed by DA_0. When the data from DA_i and DA_{i+1} has been received, the DB_i block can start decoding. The committed corrections from all blocks are added together, continuously updating the total correction.

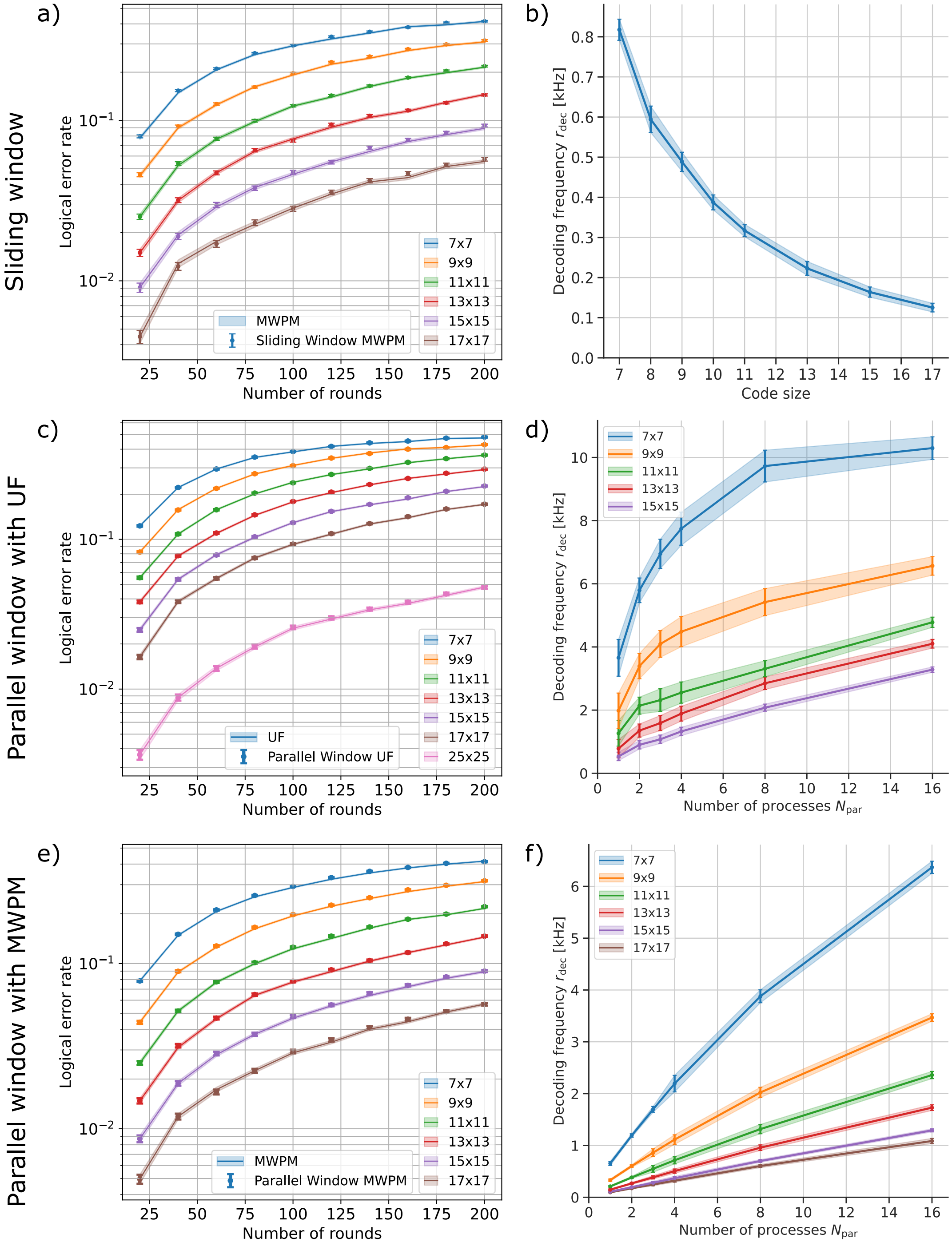

Supplementary Figure 3. Logical error rate and decoding frequency on rotated planar code using sliding window MWPM decoder, and parallel window decoder with union-find under phenomenological Pauli noise with 2% physical error rate. (a) Logical error rates as a function of the number of rounds of syndrome extraction for different code sizes for the global MWPM (lines), and using the sliding window MWPM decoder (points). (b) The decoding frequency as a function of the code size d for square rotated planar codes using a sliding window MWPM decoder. (c) Logical error rates as a function of the number of rounds for global UF (lines) and using the parallel window algorithm with UF inner decoder (points). (d) The decoding frequency as a function of the number of decoding processes for the parallel window UF algorithm. (c) Logical error rates as a function of the number of rounds for global MWPM (lines) and using the parallel window algorithm with MWPM inner decoder (points). (d) The decoding frequency as a function of the number of decoding processes for the parallel window MWPM algorithm. Where the error bars are not visible, they are smaller than the marker size. Here we plot the decoding frequency r_{dec}, therefore the rate of syndrome processing is $r_{\mathrm{proc}} = r_{\mathrm{dec}}(d^2 - 1)$. (e) is the same as (c) but with MWPM as the inner decoder. (f) is the same as (d) but with MWPM as the inner decoder.

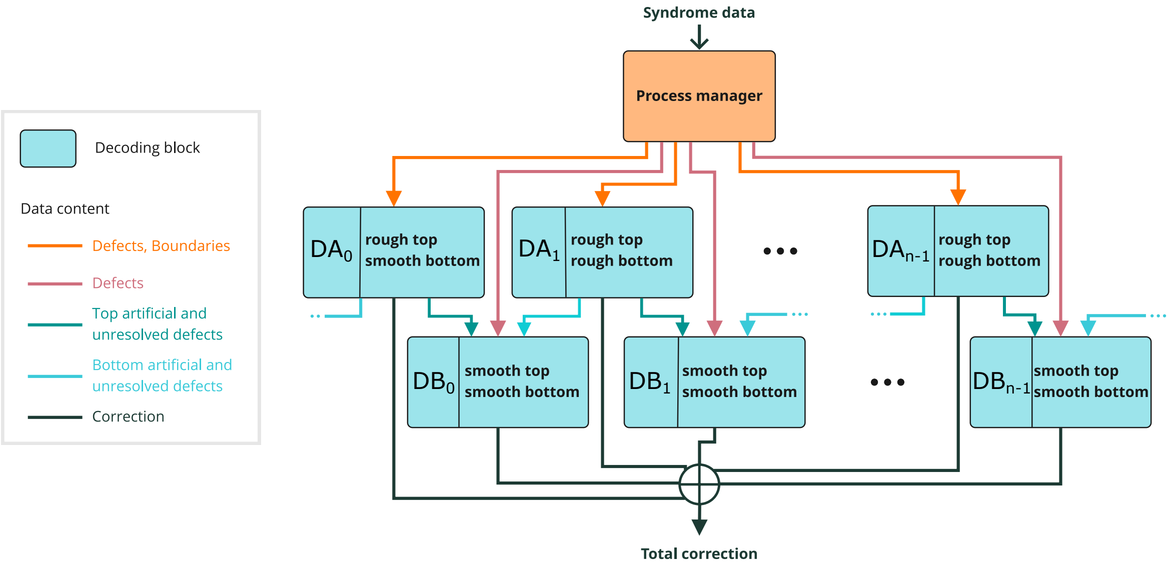

Supplementary Figure 4. Parallel window decoding pipeline. The content of the data lines is colour-coded and described in the legend on the left. Each decoding block implements a matching algorithm on $3d$ rounds with specified time boundaries. The process manager can control the time boundaries of DA_i blocks to match the global initial and final rounds. The blocks are connected cyclically as the line going from DA_0 to the left is connected to the line to DB_{n-1} coming from the right.

[1] C. Horsman, A. G. Fowler, S. Devitt, and R. Van Meter, Surface code quantum computing by lattice surgery, New Journal of Physics **14**, 123011 (2012).
[2] A. G. Fowler and C. Gidney, Low overhead quantum computation using lattice surgery, arXiv preprint arXiv:1808.06709 (2018).
[3] D. Litinski, A game of surface codes: Large-scale quantum computing with lattice surgery, Quantum **3**, 128 (2019).
[4] C. Chamberland and E. T. Campbell, Circuit-level protocol and analysis for twist-based lattice surgery, Physical Review Research **4**, 023090 (2022).
[5] C. Chamberland and E. T. Campbell, Universal quantum computing with twist-free and temporally encoded lattice surgery, PRX Quantum **3**, 010331 (2022).
[6] H. Bombín, An introduction to topological quantum codes, arXiv preprint arXiv:1311.0277 (2013).
[7] A. Kubica and M. E. Beverland, Universal transversal gates with color codes: A simplified approach, Phys. Rev. A **91**, 032330 (2015).
[8] P. Das, C. A. Pattison, S. Manne, D. Carmean, K. Svore, M. Qureshi, and N. Delfosse, A scalable decoder micro-architecture for fault-tolerant quantum computing, arXiv preprint arXiv:2001.06598 (2020).
[9] O. Higgott and C. Gidney, Sparse blossom: Correcting a million errors per core second with minimum-weight matching, arXiv preprint arXiv:2303.15933 (2023).
